# Supplementary material for: Mutations in PCYT2 disrupt etherlipid biosynthesis and cause a complex hereditary spastic paraplegia
Source: Brain. 2019 Oct 22;142(11):3382–97. doi: 10.1093/brain/awz291 (PMC6821184; doi:10.1093/brain/awz291)
Supplement: awz291_Supplementary_Data [file awz291_supplementary_data.zip › awz291-Suppl_data/Supplementary_Data4.zip!/Supplementary information 3 - fibroblast lipidomics.zip!/Fibroblast lipidomics/Patient1 vs Patient2/Lipidomics.pdf]

CORE FACILITY METABOLOMICS
Laboratory Genetic Metabolic Diseases
Meibergdreef 9
1105 AZ Amsterdam

Frédéric Vaz +31 20 5662890
Michel van Weeghel +31 20 5661918

October 29, 2018

LIPIDOMICS ANALYSIS REPORT

Lipidomics analysis performed for:
Frédéric Vaz

Project manager and contact AMC:
Michel van Weeghel
m.vanweeghel@amc.nl

Lipidomics analysis was performed by Martin Vervaart
Bioinformatics analysis was performed by Angela Luyf, Michel van Weeghel

Report version 2018.002

About the report

This is automatically generated report. In this report the lipidomics analysis and the performed statistical analysis are explained. The report also contains a visual summary of the data which was provided in this folder. For further questions regarding the data please do not hesitate and contact the project manager.

All the individual plots and data analysis can be found in the folder "Statistical plots and data". These plots include a correlation matrix (based on AOE), several heatmaps where lipids are sorted using different parameters (eg. p-value and VIP-score), PCA analysis, (O)PLS-DA analysis, random forest calculations, volcano plots, separate lipid species plots (provided in seperate folders within the "Statistical plots and data" folder) and an excel sheet ("Statistical data.xlsx") containing all the statistical analysis done on the data set.

About the lipidomics analysis

Method

Lipidomics analysis was performed as described (https://doi.org/10.1194/jlr.M067470). The HPLC system consisted of an Ultimate 3000 binary HPLC pump, a vacuum degasser, a column temperature controller, and an auto sampler (Thermo Scientific, Waltham, MA, USA). The column temperature was maintained at 25 °C. The lipid extract was injected onto a "normal phase column" LiChrospher 2x250-mm silica-60 column, 5 μm particle diameter (Merck, Darmstadt, Germany) and a "reverse phase column" Acquity UPLC HSS T3, 1.8 μm particle diameter (Waters, Milford Massachusetts, USA). A Q Exactive Plus Orbitrap (Thermo Scientific) mass spectrometer was used in the negative and positive electrospray ionization mode. Nitrogen was used as the nebulizing gas. The spray voltage used was 2500 V, and the capillary temperature was 256 °C. S-lens RF level: 50, auxiliary gas: 11, auxiliary temperature 300 °C, sheath gas: 48, sweep cone gas: 2. In both the negative and positive ionization mode, mass spectra of the lipid species were obtained by continuous scanning from m/z 150 to m/z 2000 with a resolution of 280,000 full width at half maximum (FWHM).

Identification

Identification is based on exact mass (with 3 ppm tolerance) and retention time including the relation between these two parameters taking into account the different molecular species of the lipid class (see: Herzog et al: https://doi.org/10.1194/jlr.M067470). Our identification is thus mostly tentative, the current experiment is a screen to identify interesting features/species that should be further analyzed to confirm their identity (by MS/MS, NMR or other methods).

Quantitation

Our approach is semi-quantitative at best and results cannot be directly compared to other measurements that have been done in separate experiments. At least one internal standard per major class is used to normalize the intensities of the species of that major class. Please keep in mind that the abundance per nmol of the internal standard used could be (very) different than for other species of the same lipid class. The reported response of the lipid species (defined as the ratio between the abundance of a selected lipid species and the abundance of the internal standard) can only be used to compare the same lipid species across different samples/groups.

Interpretation

Keep in mind that the statistical significant changes could be an overestimation of the real changes between the groups. An isolated significant change in one lipid species per se is not as important as a systematic change of similar species within or across lipid classes (e.g. a shift in unsaturation or accumulation of smaller species).

Lipid species abbreviations

The glycerophospholipid abbreviations (PC, PE, etc.) are used to refer to species with one or two radyl side-chains where the structures of the side chains are indicated within parentheses in the 'Headgroup(sn1/sn2)' format (e.g. PC(34:1). By default, R stereochemistry at the C-2 carbon of glycerol and attachment of the headgroup at the sn3 position is assumed. Also, acyl chains are assumed by default. The 'O-' prefix [O] is used to indicate the presence of an alkyl ether substituent e.g. PC(O-34:1), whereas the 'P-' prefix [P] is used for the 1Z-alkenyl ether (Plasmalogen) substituent e.g. PC(P-34:1).

For molecules with opposite (S) stereochemistry at C2 of the glycerol group and attachment of the headgroup at the sn1 position, the stereochemistry specification of [S] is appended to the abbreviation. The 'Headgroup(sn3/sn2)' abbreviation format is used.

For molecules with unknown stereochemistry at the C-2 carbon of the glycerol group, the stereochemistry specification of [U] is appended to the abbreviation and the structure is drawn with C-2 stereochemistry unspecified.

ID	Common name	Abbreviation	Synonyms	Category	Main class
BMP	Lysobisphosphatidic acid	LBPA	BMP	[GP]	Glycerophosphoglycerols [GP04]
CE	Cholesteryl ester	CE		[ST]	Sterols [ST01]
Cer[d]	Ceramide	Cer[d]		[SP]	Ceramides [SP02]
CL	Cardiolipin	CL		[GP]	Glycerophosphoglycerophosphoglycerols [GP12]
DG	Diradylglycerol	DG	DAG, diacylglycerol	[GL]	Diradylglycerols [GL02]
Hex2Cer[d]	Dihexosylceramide	Hex2Cer	Glc2Cer; Gal2Cer	[SP]	Neutral glycosphingolipids [SP05]
HexCer[d]	Hexocylceramide	HexCer	GlcCer; GalCer	[SP]	Neutral glycosphingolipids [SP05]
LPA	Lysophosphatic acid	LPA		[GP]	Glycerophosphates [GP10]
LPC	Lysophosphatidylcholine	LysoPC	LPC	[GP]	Glycerophosphocholines [GP01]
LPC[O]	Alkyllysophosphatidylcholine	LysoPC(O)		[GP]	Glycerophosphocholines [GP01]
LPE	Lysophosphatidylethanolamine	LysoPE	LPE	[GP]	Glycerophosphoethanolamines [GP02]
LPE[O]	Alkyllysophosphatidylethanolamine	LysoPE(O)		[GP]	Glycerophosphoethanolamines [GP02]
LPG	Lysophosphatidylglycerol	LysoPG	LPG	[GP]	Glycerophosphoglycerols [GP04]
PA	Phosphatidic acid	PA		[GP]	Glycerophosphates [GP10]
PC	Phosphatidylcholine	PC		[GP]	Glycerophosphocholines [GP01]
PC[O]	Alkylphosphatidylcholine	PC(O)		[GP]	Glycerophosphocholines [GP01]
PE	Phosphatidylethanolamine	PE		[GP]	Glycerophosphoethanolamines [GP02]
PE[O]	Alkylphosphatidylethanolamine	PE(O)		[GP]	Glycerophosphoethanolamines [GP02]
PG	Phosphatidylglycerols	PG		[GP]	Glycerophosphoglycerols [GP04]
PI	Phosphatidylinositols	PI		[GP]	Glycerophosphoinositols [GP06]
PS	Phosphatidylserine	PS		[GP]	Glycerophosphoserines [GP03]
S1P	Sphingosine/Sphinganine 1-phosphate	S1P	S1P	[SP]	Sphingoid bases [SP01]
SM[d]	Sphingomyelin/Ceramide phosphocholines	SM[d]		[SP]	Phosphosphingolipids [SP03]
SM[t]	Hydroxysphingomyelin	SM[t]		[SP]	Phosphosphingolipids [SP03]
SPH	Sphingosine, Sphinganine	SPH		[SP]	Sphingoid bases [SP01]
TG	Triradylglycerols	TG	TAG, triacylglycerol	[GL]	Triradylglycerols [GL03]
TG[O]	Alkyldiradylglycerol	TG[O]	alkyldiacylglycerol	[GL]	Triradylglycerols [GL03]

Table version 2018.002

The PCA and (O)PLS-DA analysis

Principal component analysis (PCA) is a statistical procedure that uses an orthogonal transformation to convert a set of observations of possibly correlated variables into a set of values of linearly uncorrelated variables called principal components. The number of distinct principal components is equal to the smaller of the number of original variables or the number of observations minus one. This transformation is defined in such a way that the first principal component has the largest possible variance (that is, accounts for as much of the variability in the data as possible), and each succeeding component in turn has the highest variance possible under the constraint that it is orthogonal to the preceding components. The resulting vectors are an uncorrelated orthogonal basis set. PCA is sensitive to the relative scaling of the original variables.

(**Orthogonal**) **Partial least squares regression** (PLS regression) is a statistical method that bears some relation to principal components regression; instead of finding hyperplanes of maximum variance between the response and independent variables, it finds a linear regression model by projecting the predicted variables and the observable variables to a new space. PLS is used to find the fundamental relations between two matrices (X and Y), i.e. a latent variable approach to modeling the covariance structures in these two spaces. A PLS model will try to find the multidimensional direction in the X space that explains the maximum multidimensional variance direction in the Y space. PLS regression is particularly suited when the matrix of predictors has more variables than observations, and when there is multicollinearity among X values. By contrast, standard regression will fail in these cases (unless it is regularized).

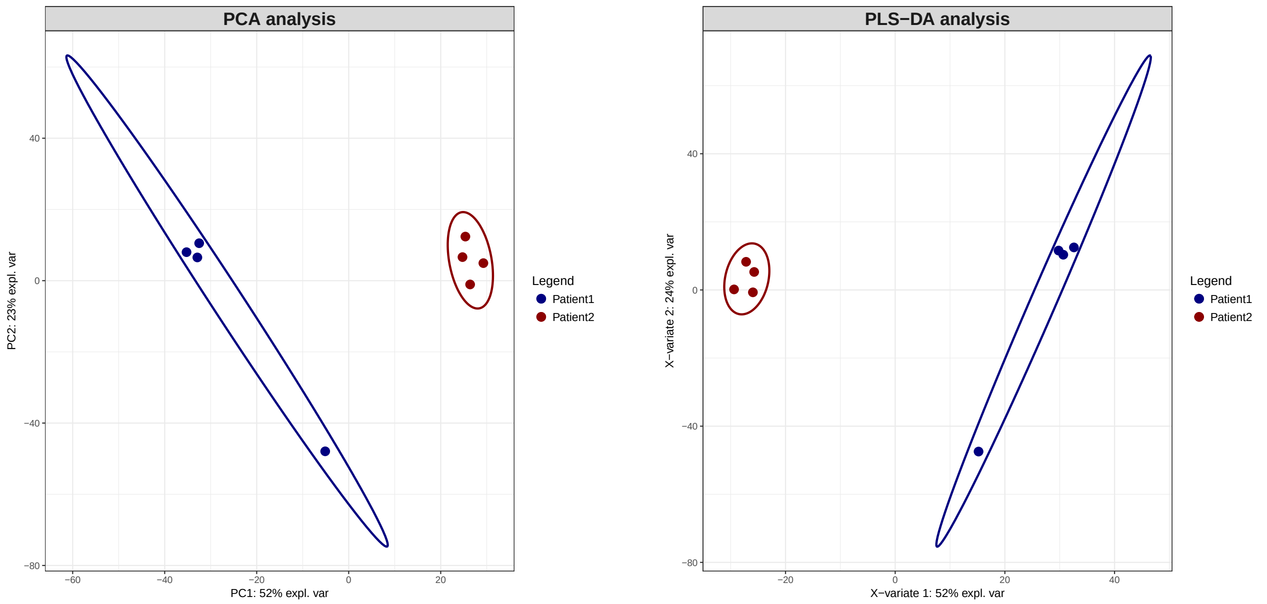

(a) PCA analysis

(b) (O)PLS-DA analysis

Figure 1: Results from the (a) PCA and (b) (O)PLS-DA analysis

Output of the PCA and (O)PLS-DA analysis

PCA analysis output:

```
1) Data set:
8 samples x 1696 variables
0 (0%) NAs
0 excluded variables (near zero variance)
standard x scaling
Summary of the 3 increasing variance spaced raw variables:
   TG(72:1)          TG(61:10)         CE(22:6)
 Min.   :0.01629   Min.   :0.4976   Min.   : 2206
 1st Qu.:0.02111   1st Qu.:0.5950   1st Qu.: 2668
 Median :0.02377   Median :1.1519   Median : 5800
 Mean   :0.02386   Mean   :1.7564   Mean   : 9881
 3rd Qu.:0.02655   3rd Qu.:3.2622   3rd Qu.:19496
 Max.   :0.03112   Max.   :3.5029   Max.   :20858

2) Model: PCA
Correlations between variables and first 2 components:
                 p1 cor_p1
CE(20:4)    -0.034  -1.00
CE(28:5)    -0.033  -1.00
CE(26:5)    -0.033  -1.00
PE(O-34:2)   0.033   0.99
LPE(O-18:1)  0.033   0.99
PE(O-36:2)   0.033   0.99
     R2X R2X(cum) Iter.
p1 0.524    0.524     0
```

(O)PLS-DA analysis output:

```
1) Data set:
8 samples x 1696 variables and 1 response
0 (0%) NAs
0 excluded variables (near zero variance)
standard x and y scaling
Summary of the 3 increasing variance spaced raw variables:
   TG(72:1)          TG(61:10)         CE(22:6)
 Min.   :0.01629   Min.   :0.4976   Min.   : 2206
 1st Qu.:0.02111   1st Qu.:0.5950   1st Qu.: 2668
 Median :0.02377   Median :1.1519   Median : 5800
 Mean   :0.02386   Mean   :1.7564   Mean   : 9881
 3rd Qu.:0.02655   3rd Qu.:3.2622   3rd Qu.:19496
 Max.   :0.03112   Max.   :3.5029   Max.   :20858

2) Model: OPLS-DA
Correlations between variables and first 2 components:
                h1     o1 cor_h1 cor_o1
CE(18:2)    -0.034     NA  -1.00     NA
CE(20:2)    -0.034     NA  -1.00     NA
DG(30:0)    -0.034     NA  -1.00     NA
PS(O-36:2)   0.034     NA   0.98     NA
PS(O-36:1)   0.034     NA   0.99     NA
PS(O-34:1)   0.034     NA   1.00     NA
PC(33:0)        NA -0.046     NA  -0.93
PC(44:11)       NA -0.046     NA  -0.93
PC(O-34:0)      NA -0.044     NA  -0.88
LPC(O-24:1)     NA  0.049     NA   0.98
LPC(O-13:0)     NA  0.049     NA   0.98
PC(48:6)        NA  0.049     NA   0.99
      R2X R2X(cum)    R2Y R2Y(cum)    Q2 Q2(cum) Signif.
p1  0.495    0.495 0.9670   0.9670 0.890   0.890      R1
o1  0.258    0.753 0.0309   0.0309 0.057   0.057      R1
sum    NA    0.753     NA   0.9980    NA   0.947    <NA>
```

VIP-score

Variable Importance Projection (VIP) scores estimate the importance of each variable in the projection used in a PLS model and is often used for variable selection. A variable with a VIP Score close to or greater than 1 (one) can be considered important in given model. Variables with VIP scores significantly less than 1 (one) are less important and might be good candidates for exclusion from the model.

It should be noted that the nature of the VIP calculation is such that when the model is rebuilt, new variables will always be below the threshold so this approach does not lend itself to repeated variable exclusion.

The input is a PLS model structure (model) or the outputs from SIMPLS or NIPPLS. The output (vip scores) is a set of column vectors equal in length to the number of variables included in the model. It contains one column of VIP scores for each column of the original calibration y-block. In the case of multiple columns the values on each row should be compared logically if any is >1, or use a max operation, to decide which rows (original variables) are important (http://dx.doi.org/10.1016/j.chemolab.2004.12.011).

Metabolite	P_Value	HOLM	FDR	Log2_fold_change	VIP_score
CE(21:4)	0.0000	0.0000	0.0000	-2.4620	1.4010
CE(18:2)	0.0000	0.0000	0.0000	-2.0650	1.4010
CE(20:2)	0.0000	0.0000	0.0000	-2.8250	1.4000
DG(30:0)	0.0000	0.0000	0.0000	-1.0820	1.3980
CE(25:3)	0.0000	0.0100	0.0010	-3.2520	1.3980
PS(O-34:1)	0.0000	0.0390	0.0010	1.5020	1.3980
CE(26:3)	0.0000	0.0010	0.0000	-3.3530	1.3970
CE(27:4)	0.0000	0.0030	0.0000	-3.0090	1.3970
CE(24:2)	0.0000	0.0010	0.0000	-2.7790	1.3960
PC(34:3)	0.0000	0.0100	0.0010	-0.5620	1.3960
SM(d40:3)	0.0000	0.0590	0.0020	-0.3750	1.3950
PS(40:6)	0.0000	0.3200	0.0050	-0.9040	1.3950
CE(25:4)	0.0000	0.1210	0.0030	-3.1220	1.3940
SM(d42:4)	0.0000	0.0430	0.0010	-0.6540	1.3940
CE(19:3)	0.0000	0.0610	0.0020	-2.7690	1.3930
CE(25:2)	0.0000	0.0020	0.0000	-2.5110	1.3930
CE(26:2)	0.0000	0.0010	0.0000	-2.8290	1.3930
CE(16:3)	0.0000	0.0350	0.0010	-3.3860	1.3930
CE(26:1)	0.0000	0.0030	0.0000	-2.5620	1.3920
CE(19:1)	0.0000	0.0290	0.0010	-2.0050	1.3920
CE(23:2)	0.0000	0.0020	0.0000	-2.5340	1.3920
PS(O-36:1)	0.0000	0.0650	0.0020	1.8340	1.3920
CE(21:2)	0.0000	0.0510	0.0020	-2.5890	1.3910
CE(22:2)	0.0000	0.1700	0.0030	-3.1270	1.3890
CE(19:2)	0.0000	0.0400	0.0010	-2.4820	1.3890
HexCer(d32:1)	0.0000	0.0240	0.0010	-1.2420	1.3890
CE(16:0)	0.0000	0.0070	0.0010	-1.7010	1.3880
CE(27:1)	0.0000	0.0090	0.0010	-1.9940	1.3880
PC(33:2)	0.0000	0.0110	0.0010	-0.6350	1.3860
CE(18:1)	0.0000	0.0110	0.0010	-2.2170	1.3860
HexCer(d43:2)	0.0000	0.0250	0.0010	-0.7110	1.3850
CE(18:0)	0.0000	0.0090	0.0010	-2.1610	1.3850
PS(38:3)	0.0000	0.2000	0.0040	-0.6540	1.3840
PC(O-39:7)	0.0000	0.0330	0.0010	-0.5450	1.3840
DG(30:1)	0.0000	0.5430	0.0070	-1.0590	1.3830
CE(24:3)	0.0000	0.5950	0.0070	-3.1060	1.3820
CE(25:1)	0.0000	0.0130	0.0010	-2.0790	1.3820
PS(36:3)	0.0000	0.0250	0.0010	-0.7220	1.3810
CE(29:5)	0.0010	1.0000	0.0100	-5.5220	1.3810
PS(40:5)	0.0000	0.4390	0.0060	-1.0420	1.3810
PE(O-36:1)	0.0000	0.0230	0.0010	1.7960	1.3800
SM(t34:1)	0.0010	0.8750	0.0090	-1.1890	1.3800
SM(t33:1)	0.0000	0.0190	0.0010	-0.7700	1.3800
CE(21:5)	0.0010	1.0000	0.0100	-2.6890	1.3790
LPC(19:0)	0.0000	0.0700	0.0020	-0.4580	1.3790
CE(28:3)	0.0000	0.5020	0.0070	-3.3080	1.3790
CE(23:4)	0.0000	0.7660	0.0080	-3.8920	1.3780
SM(t34:0)	0.0010	0.8190	0.0090	-0.8570	1.3780
CE(27:3)	0.0010	0.9160	0.0090	-3.3370	1.3780
PS(O-36:2)	0.0000	0.0840	0.0020	1.4960	1.3770

Table 1: Top 50 most important changed lipids species in the data set (sorted on the VIP-score)

Heatmap analysis

A **heatmap** is a graphical representation of data where the individual values contained in the sample set are represented as colors. The heatmap is created by calculating the Z score of each individual sample in a row (metabolite). Z = (X (value of the individual sample) - μ (average of the row)) / σ (standard deviation of the row). The lipids in the heatmap plotted here are the 50 most important changed (sorted on their VIP-score) between the different groups. The VIP-score is explained on page 4.

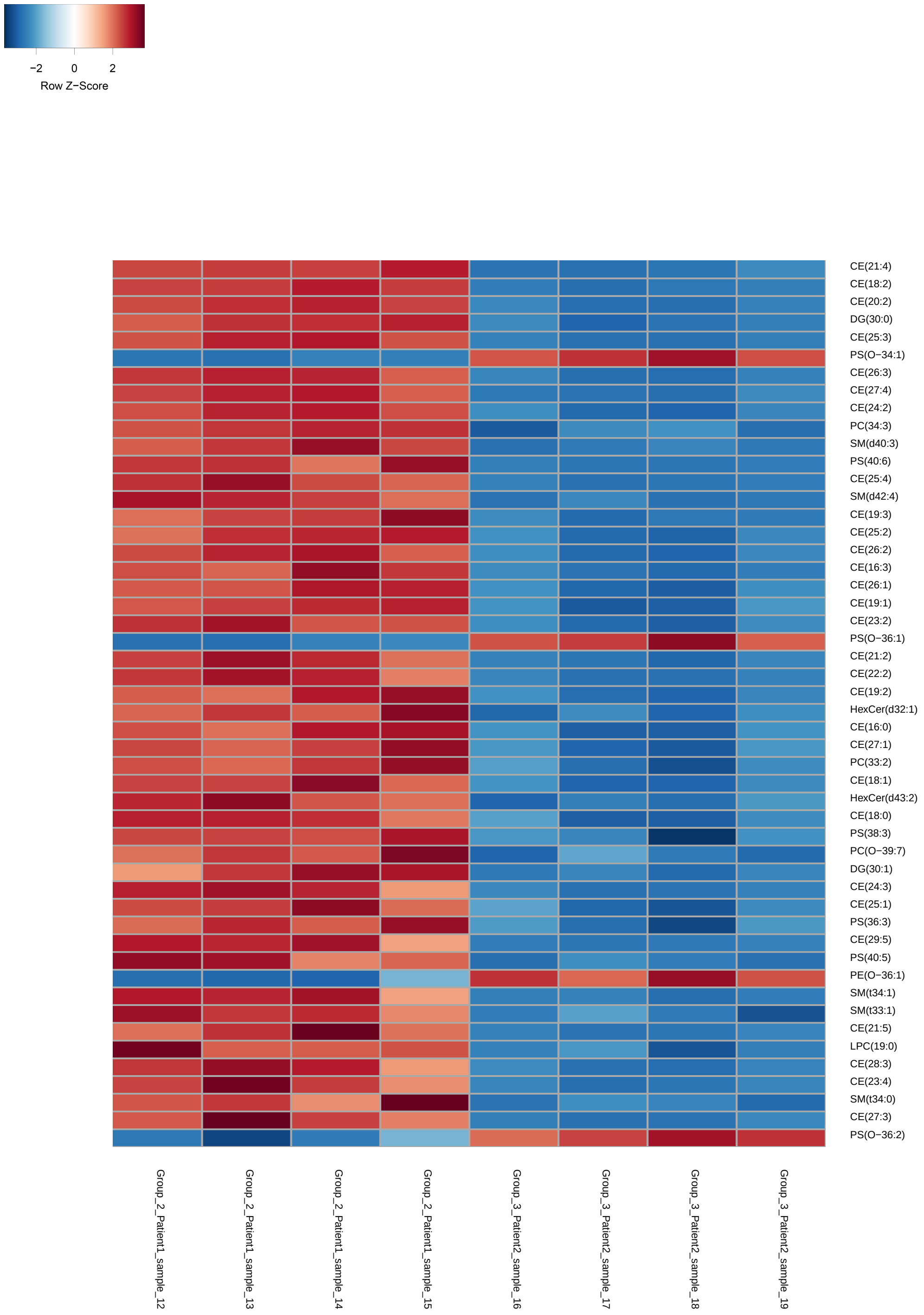

Figure 2: Heatmap with the 50 most important changes (sorted on their VIP-score) between the different groups

Random forest analysis

random forest is an ensemble learning method for classification, regression and other tasks, that operate by constructing a multitude of decision trees. Random forests do this in two ways: 1) by bagging, for bootstrap aggregating. Bagging takes a randomized sample of the rows in your training set, with replacement and 2) instead of looking at the entire pool of available variables, Random Forests take only a subset of them, typically the square root of the number available.

Because the random forest process has two sources of randomness we need to set a random seed number which is set to 415. The number itself isn't important, we just have to ensure to use the same seed number each time so that the same random numbers are generated inside the Random Forest function. Furthermore, random Forests doesn't just waste "out-of-bag" (OOB) observations, it uses them to see how well each tree performs on unseen data. It's almost like a bonus test set to determine your model's performance on the fly.

There's two types of importance measures shown in the graph below. The accuracy one tests to see how worse the model performs without each variable, so a high decrease in accuracy would be expected for very predictive variables. The Gini one digs into the mathematics behind decision trees, but essentially measures how pure the nodes are at the end of the tree. Again it tests to see the result if each variable is taken out and a high score means the variable was important (http://trevorstephens.com/kaggle-titanic-tutorial/r-part-5-random-forests/).

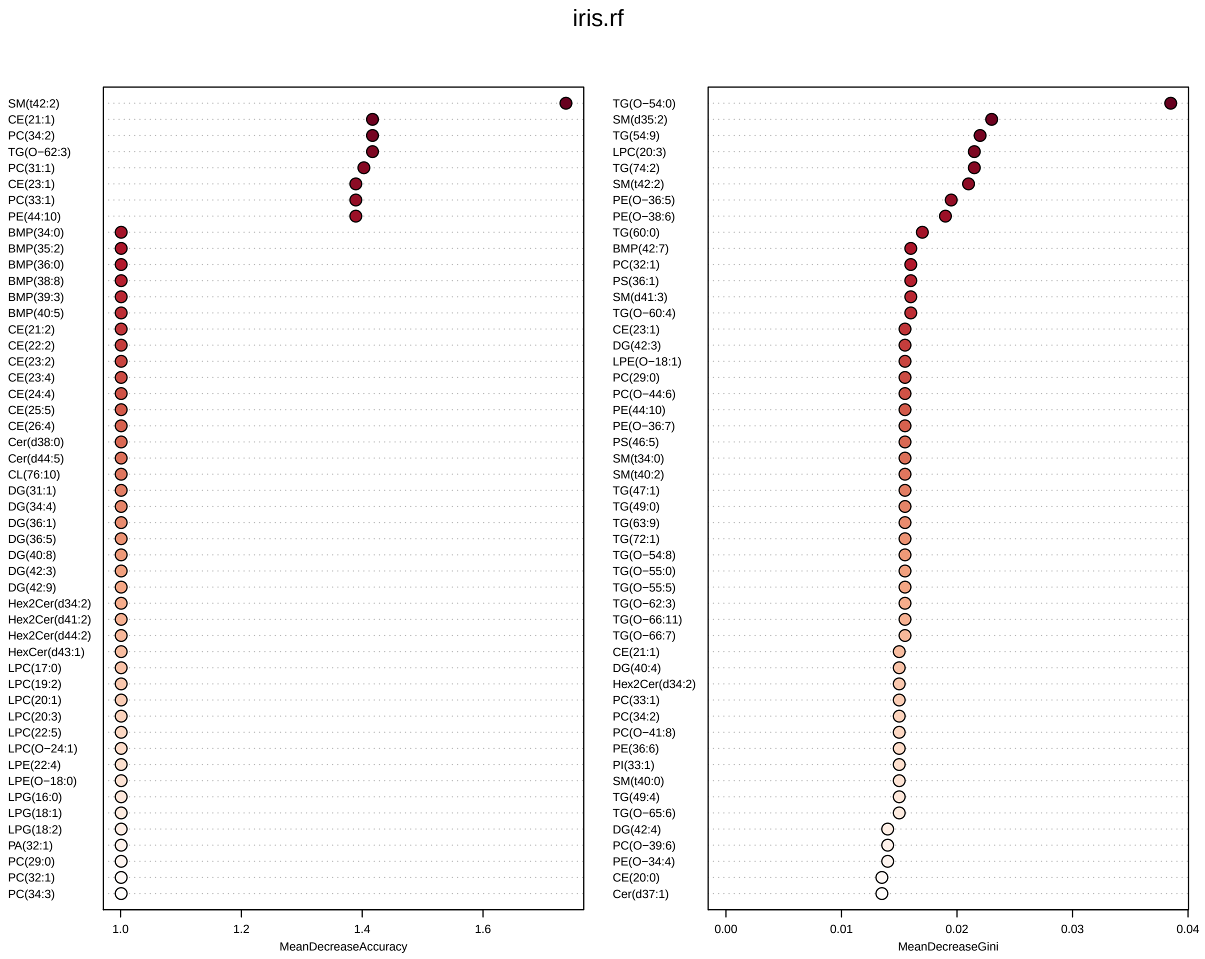

Figure 3: Random forest analysis with the random number set on 415

```
Call:
 randomForest(x = RandomForestData, y = grpsE, importance = TRUE)
               Type of random forest: classification
                     Number of trees: 500
No. of variables tried at each split: 41

        OOB estimate of  error rate: 12.5%
Confusion matrix:
         Patient1 Patient2 class.error
Patient1        3        1        0.25
Patient2        0        4        0.00
```

Volcano plot analysis

The **volcano plot** is a type of scatter-plot that is used to quickly identify changes in large data sets composed of replicate data. It plots significance (-$\log_{10}$(p-value)) versus ($\log_2$(fold change)) on the y and x axis, respectively. These plots are increasingly common in omic experiments such as genomics, proteomics, metabolomics and **lipidomics** where one often has a list of many thousands of replicate data points between two conditions and one wishes to quickly identify the most meaningful changes (https://doi.org/10.1186/gb-2003-4-4-210).

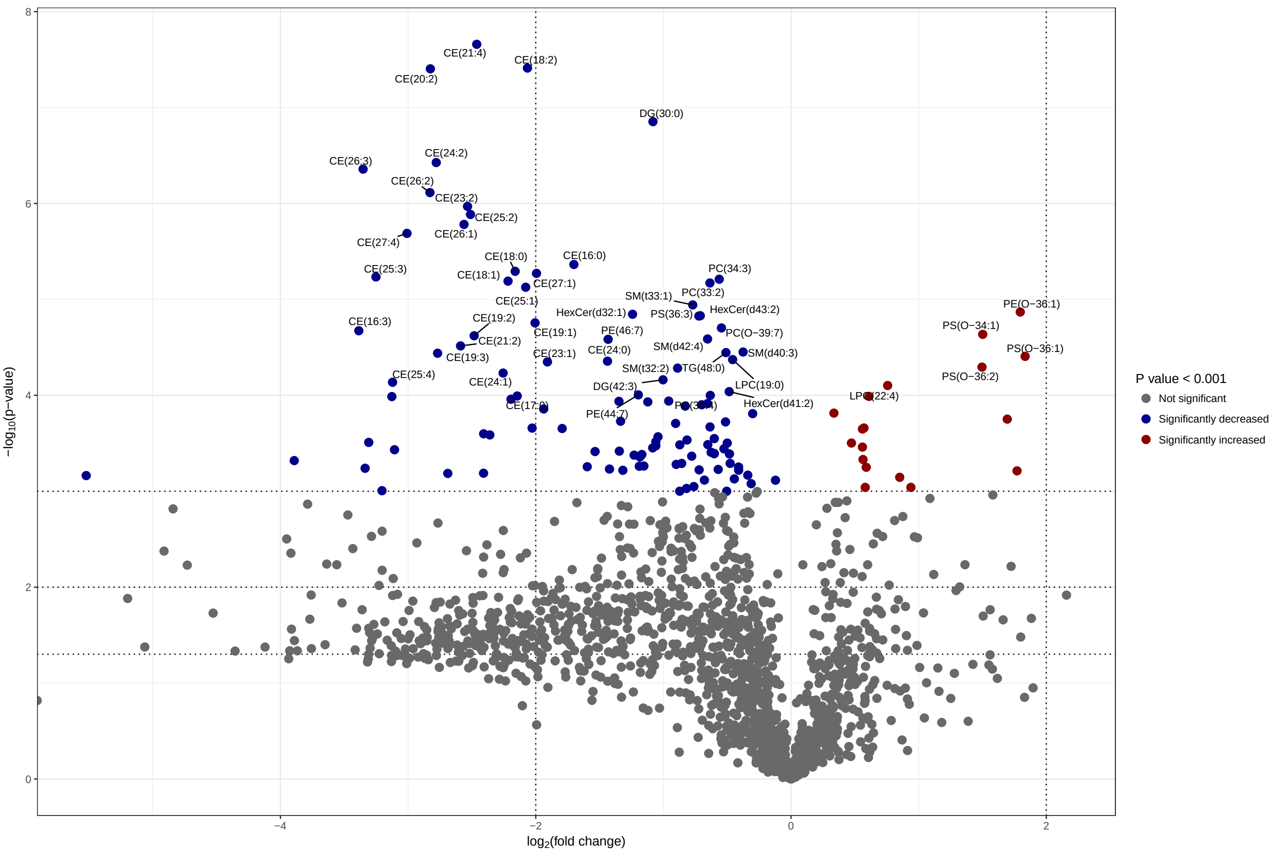

Figure 4: Volcano plot depicting the lipidomics data. Significance cutoff is shown in the legend of the volcano plot. The 50 most changed lipid species based on p-value are labeled. The three horizontal dotted lines indicate p-value 0.05, 0.01 and 0.001, respectively. The two vertical dotted lines indicate $\log_2$(fold change) -2 and 2, respectively.

Correlation matrix

A **correlation matrix** is a table showing correlation coefficients between lipids. Each random variable (X_i) in the table is correlated with each of the other values in the table (X_j). This allows you to see which pairs have the highest correlation.

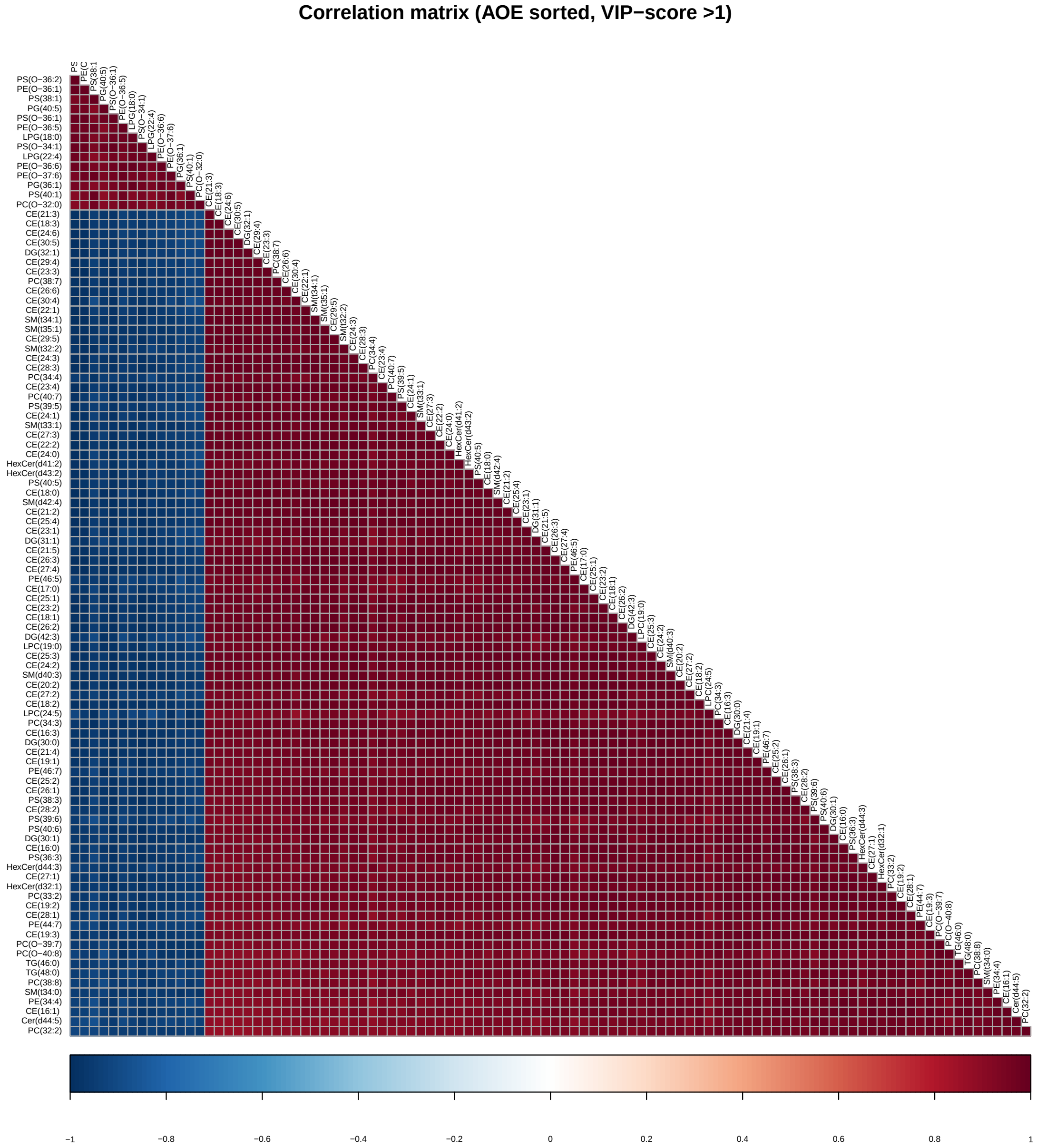

Figure 5: Correlation matrix (Pearson) sorted on the angular order of eigenvectors (AOE)

Lipid species analysis

The datafolder "Statistical analysis and plots" contain folders of all the separate **lipid species** measured in this analysis. These separate folders contain boxplots, heatmaps and volcano plots which are sorted on chain length and saturation state of the lipid species. The folder also contains a random forest analysis on the specific lipid species. The different boxplots contain information about outliers, the median, the interquartile range (IQR) and the maximums and minimums together with the average, standard deviation and the individual data points. The boxplot layout is explained in figure 6.

By using this analysis and observing these plots, trends in saturation changes and total amounts of a certain lipid class can be seen. In this report we depict the totals of the lipid species as a summary in two distinct boxplots. The first boxplot contains all the lipid species in a separate figure (with y = total value) (figure 7). The second boxplot is depicting all species in one big boxplot (with y = $\log_{10}$ value) (figure 8). Keep in mind that the totals between the different lipid species can not be compared 1 to 1.

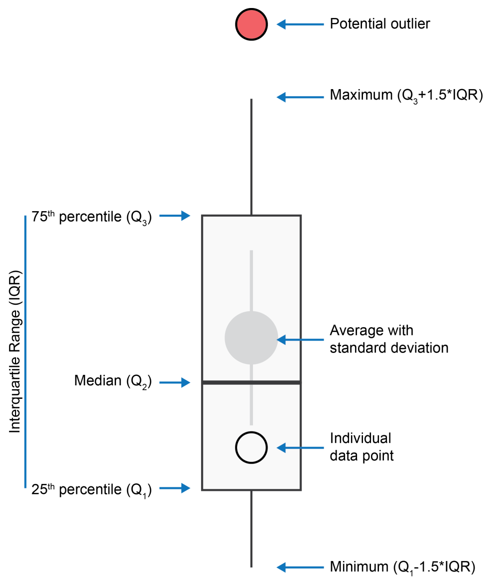

Figure 6: Boxplot layout

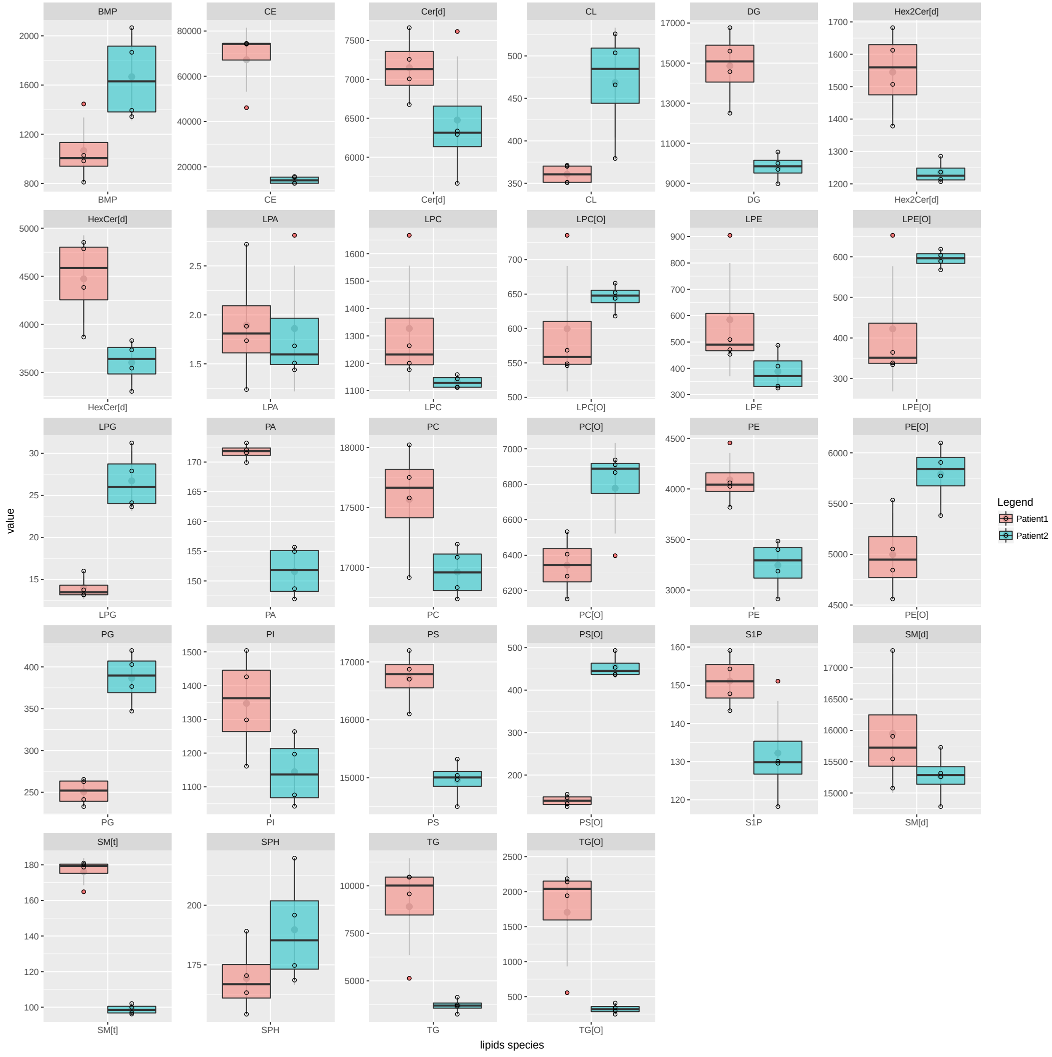

Figure 7: Lipid species totals plot (y = total value)

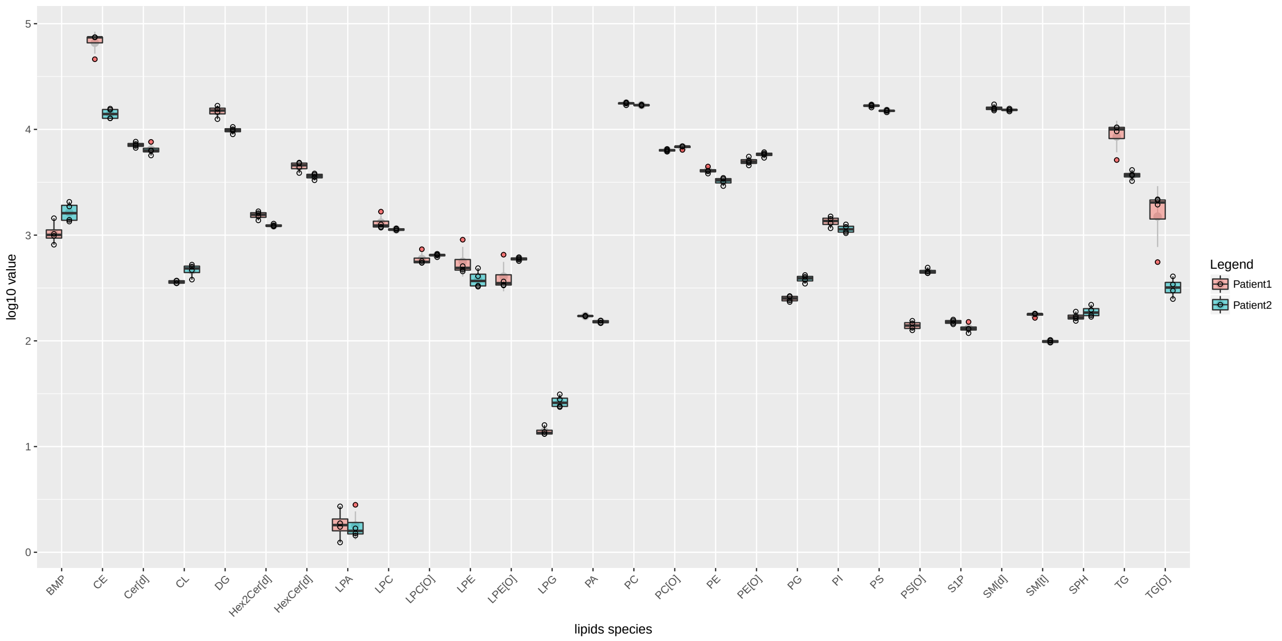

Figure 8: Lipid species summary plot all (y = log10 value)
